# Hepatocyte specific expression of an oncogenic variant of β-catenin results in cholestatic liver disease

**DOI:** 10.18632/oncotarget.13521

**Published:** 2016-11-23

**Authors:** Ursula J. Lemberger, Claudia D. Fuchs, Matthias Karer, Stefanie Haas, Tatjana Stojakovic, Christian Schöfer, Hanns-Ulrich Marschall, Fritz Wrba, Makoto M. Taketo, Gerda Egger, Michael Trauner, Christophr H. Österreiche

**Affiliations:** ^1^ Institute of Pharmacology, Medical University of Vienna, Vienna, Austria; ^2^ Clinical Institute of Pathology, Medical University of Vienna, Vienna, Austria; ^3^ Hans Popper Laboratory for Molecular Hepatology, Department of Internal Medicine, Medical University of Vienna, Vienna, Austria; ^4^ Clinical Institute of Medical and Chemical Laboratory Diagnostics, Medical University of Graz, Graz, Austria; ^5^ Department of Cell and Developmental Biology, Medical University of Vienna, Vienna, Austria; ^6^ Department of Molecular and Clinical Medicine, Sahlgrenska Academy, University of Gothenburg, Gothenburg, Sweden; ^7^ Division of Experimental Therapeutics, Graduate School of Medicine, Kyoto University, Kyoto, Japan

**Keywords:** β-catenin, bile acids, cholestasis, biliary fibrosis, liver cancer

## Abstract

**Background:**

The Wnt/β-catenin signaling pathway plays a crucial role in embryonic development, tissue homeostasis, wound healing and malignant transformation in different organs including the liver. The consequences of continuous β-catenin signaling in hepatocytes remain elusive.

**Results:**

Livers of *Ctnnb1^CA hep^* mice were characterized by disturbed liver architecture, proliferating cholangiocytes and biliary type of fibrosis. Serum ALT and bile acid levels were significantly increased in *Ctnnb1^CA hep^* mice. The primary bile acid synthesis enzyme *Cyp7a1* was increased whereas *Cyp27* and *Cyp8b1* were reduced in *Ctnnb1^CA hep^* mice. Expression of compensatory bile acid transporters including *Abcb1*, *Abcb4*, *Abcc2* and *Abcc4* were significantly increased in *Ctnnb1^CA hep^* mice while *Ntcp* was reduced. Accompanying changes of bile acid transporters favoring excretion of bile acids were observed in intestine and kidneys of *Ctnnb1^CA hep^* mice. Additionally, disturbed bile acid regulation through the FXR-FGF15-FGFR4 pathway was observed in mice with activated β-catenin.

**Materials and Methods:**

Mice with a loxP-flanked exon 3 of the *Ctnnb1* gene were crossed to *Albumin-Cre* mice to obtain mice with hepatocyte-specific expression of a dominant stable form of β-catenin (*Ctnnb1^CA hep^* mice). *Ctnnb1^CA hep^* mice were analyzed by histology, serum biochemistry and mRNA profiling.

**Conclusions:**

Expression of a dominant stable form of β-catenin in hepatocytes results in severe cholestasis and biliary type fibrosis.

## INTRODUCTION

The Wnt/β-catenin signaling pathway is an evolutionary preserved system, which plays a crucial role in embryonic development, tissue homeostasis, wound healing and malignant transformation in different organs including the liver [1–3]. β-catenin forms a cytoplasmatic complex with axin, adenomatous polyposis coli (APC) and glycogen synthase kinase-3β (GSK-3β) and is the key mediator of the canonical Wnt signaling pathway. In the absence of Wnt stimulation, GSK-3β is active and phosphorylates different serine and threonine residues (S33, S37, T41 and S45) within exon 3 of the *Ctnnb1* gene, leading to proteasomal degradation of β-catenin. Canonical Wnt signaling results in inactivation of GSK-3β and accordingly leads to inhibition of ubiquitin-mediated degradation of β-catenin. This leads to cytoplasmic accumulation and subsequent nuclear translocation of β-catenin, where it replaces Groucho from T-cell factors (TCFs). β-catenin then forms a transcriptional activation complex following recruitment of transcriptional co-activators and histone modifiers and induces expression of *cyclinD1, c-myc, glutamine synthetase* and other target genes [1].

Genetic deletion of β-catenin in mice results in 100% lethality at embryonic day E9.5 due to defects in anterior-posterior development [4]. The subsequent generation of mice with a conditional allele of β-catenin has allowed investigating the role of β-catenin in a cell type specific manner [5]. Mice with hepatocyte-specific deletion of β-catenin (*Ctnnb1^Δhep^*) have significantly improved our understanding of the role of β-catenin in liver biology and liver disease. In this respect, it was demonstrated that β-catenin is temporally regulated during normal liver development and controls hepatic morphogenesis and zonation of the liver [6–10]. Hepatocyte-specific deletion of β-catenin results in bile canalicular abnormalities, bile secretory defects and intrahepatic cholestasis [11]. Bile acid synthesis largely depends on the first and rate-liming enzyme CYP7A1 and is regulated by feedback mechanisms involving the Farnesoid X receptor (FXR) [12]. A complex network of transcription factors, bile acid forming and modifying enzymes and bile acid transporters and regulates formation and secretion of bile acids into the small intestine, their re-absorption and transport to the liver or their excretion via feces and urine. These include members of the ATP-binding cassette (ABC) transporter family such as ABCB11, the main transporter for bile acids in hepatocytes. Additionally, there are compensatory transporters such as ABCC2 and ABCB1. Hepatocytes are also equipped with transporters at their basolateral membrane, which allows them to shuttle bile acids into the blood of the systemic circulation via ABCC4 enabling renal clearance [13]. Morevoer, the Na+ co-transporter polypeptide (NTCP) mediates the re-uptake of bile acids reaching the liver from enterohepatic circulation [12].

Furthermore, *Ctnnb1^Δhep^* mice also display delayed liver regeneration following partial hepatectomy and are susceptible to the development of steatohepatitis in response to metabolic stress (MCD diet) and alcohol [9, 14–18]. In contrast, *Ctnnb1^Δhep^* mice are protected from acetaminophen induced hepatotoxicity due to lack of CYP2E1 expression, a key enzyme in the metabolism of acetaminophen and responsible for the formation of the toxic metabolite N-acetyl-p-benzoquinone imine which causes hepatocyte necrosis [19].

Somatic mutations of the β-catenin gene are frequent in mouse and human hepatocellular carcinomas (HCC) [20–25]. In a recent study it was demonstrated that the Wnt/β-catenin pathway is the most frequently altered signaling pathway in patients with HCC [26]. Somatic mutations in *CTNNB1* were observed in 32.8% of patients typically affecting serine and threonine residues encoded by exon 3 of the *CTNNB1* gene. Furthermore, inactivating mutations in *AXIN1* and *APC* leading to activation of β-catenin were reported in 15.2% and 1.6% of patients respectively [27].

These findings suggest that mutations occurring in genes involved in the Wnt/β-catenin signaling pathway affect ∼50% of patients with HCC. It was also reported that HCC harboring somatic missense mutations in exon 3 of *CTNNB1* exhibit a histologically more aggressive phenotype [24]. However, adenovirus-mediated expression of a dominant stable mutant of β-catenin does not result in tumorigenesis in mice suggesting that continuous β-catenin signaling alone is not sufficient for malignant transformation of hepatocytes [28]. The same laboratory later demonstrated that β-catenin cooperates with Ha-Ras in hepatocarcinogenesis in mice [29]. Mice expressing a serine-45 mutant, non-degradable form of β-catenin display accelerated hepatocarcinogenesis in response to diethylnitrosamine (DEN) treatment [15]. However, conditional loss of β-catenin in mice also promotes chemical hepatocarcinogenesis due to impaired handling of oxidative stress in response to DEN treatment [30].

Whereas *Ctnnb1^Δhep^* mice have significantly improved our understanding on the role of β-catenin in liver biology they do not mimic human liver disease. A significant proportion of human HCCs are characterized by somatic mutations in *CTNNB1* associated with continuous β-catenin signaling rather than loss of function.

In the present study we addressed the consequences of continuous β-catenin signaling in hepatocytes on liver biology.

## RESULTS

### Continuous β-catenin signaling leads to liver injury, bile duct proliferation and biliary type fibrosis

**Figure 1:**
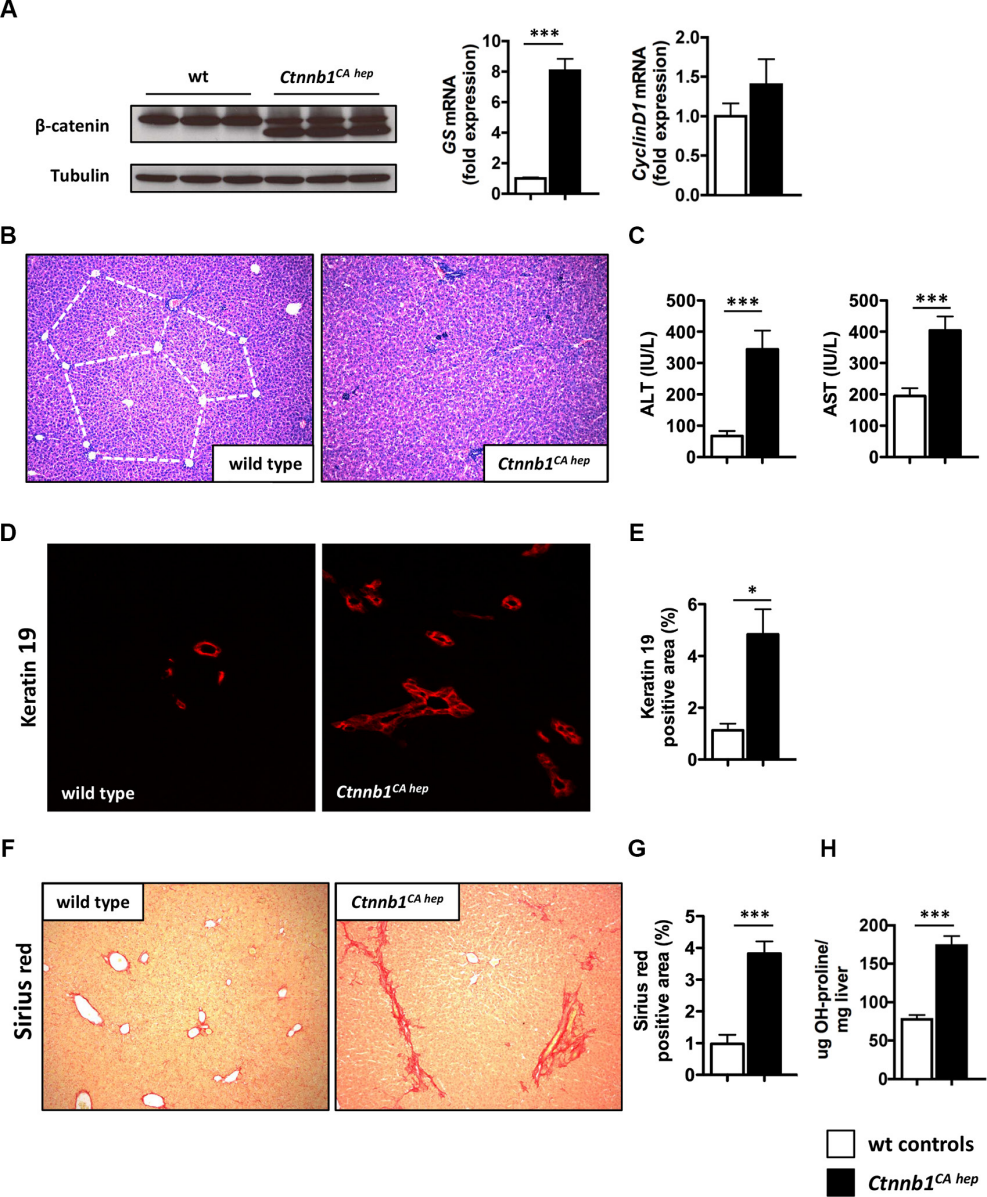
Continuous β-catenin signaling causes liver injury, bile duct proliferation and biliary type fibrosis (**A**) Western blot of livers from *Ctnnb1^CA hep^* mice and wild type (wt) controls shows expression of a truncated version of β-catenin (faster migrating band) and a lower expressed full-length version as in wt controls (left panel). mRNA expression analysis using qPCR shows highly significant induction of the β-catenin target gene glutamine synthetase (GS) and slightly induced Cyclin D1 expression (right panel). *Ctnnb1^CA hep^* mice display disturbed liver architecture (**B**) and increased serum ALT and AST levels (**C**). Immunofluorescence staining for cholangiocyte marker Keratin 19 shows a significant increase of Keratin 19 positive cells in *Ctnnb1^CA hep^* mice (**D**, **E**). Sirius red staining of wild type and *Ctnnb1 ^CA hep^* mice reveals biliary type fibrosis in β-catenin activated livers (**F**, **G**). Quantification of hepatic hydroxyproline levels confirmed increased collagen deposition in *Ctnnb1^CA hep^* livers (**H**). Morphometric quantifications were done using Image J software.

Hepatocyte specific expression of a dominant stable form of β-catenin was accomplished by crossing *Albumin-Cre* mice in which Cre recombinase is driven by the mouse *Albumin* enhancer/promoter to mice in which exon 3 of the *Ctnnb1* gene is flanked by loxP sequences, further referred to as *Ctnnb1^CA hep^*. Mice were born at the expected Mendelian ratio (Cre positive: 53.1%) and no differences with respect to sex distribution were observed. The expression of the truncated form of β-catenin was confirmed by immunoblotting of whole liver tissue of *Ctnnb1^CA hep^* mice and healthy littermates (Figure 1A). Glutamine synthetase (*Glul*) mRNA levels were 8-fold increased and Cyclin D1 (*Ccnd1*) mRNA showed a mild trend towards upregulation in *Ctnnb1^CA hep^* mice compared to Cre-negative controls indicating continuous β-catenin signaling and transcription of β-catenin target genes (Figure 1A). To investigate putative early phenotypic consequences of continuous β-catenin activation we analyzed mice at the age of 21 days upon weaning. Livers of *Ctnnb1^CA hep^* mice showed a disturbed liver architecture. Intriguingly no signs of tumor formation could be observed (Figure 1B). Ki67 staining revealed no alterations in proliferation of liver cells (Supplementary Figure S1A), however a slight increase in apoptosis of cholangiocytes was observed in livers of *Ctnnb1^CA hep^* mice as analyzed by cleaved caspase3 staining (Supplementary Figure S1B). Serum alanine aminotransferase (ALT) levels and aspartate aminotransferase (AST) were significantly increased in *Ctnnb1^CA hep^* mice (ALT: 344.4 vs. 59.6 IU/L, AST: 194.3 vs. 404.8 IU/L, *p <* 0.001, Figure 1C). However, no changes in bilirubin levels could be detected (Supplementary Figure S1C).

Immunofluorescence staining for the cholangiocyte marker Keratin 19 indicated increased numbers of cholangiocytes in *Ctnnb1^CA hep^* mice, which was confirmed by quantitation of the Keratin 19 positive areas in *Ctnnb1^CA hep^* versus wild type mice (4.8% vs. 1.1%, *p <* 0.05, Figure 1D and 1E). Evaluation of H&E stained sections confirmed these findings (Supplementary Figure S1D). Moreover, a dedifferentiation of hepatocytes in *Ctnnb1^CA hep^* mice was observed by immunofluorescence double staining, where some HNF4 positive hepatocytes also express Keratin 19 (Supplementary Figure S1E). “Reactive” cholangiocytes as observed in *Ctnnb1^CA hep^* mice are a hallmark of cholestatic liver injury. In this respect, cholestasis can lead to scarring as a consequence of a chronic wound-healing response. Accordingly, we observed a biliary type of fibrosis in *Ctnnb1^CA hep^* mice (Figure 1F). Morphometric quantification of Sirius red stained sections indicated significantly increased collagen deposition in *Ctnnb1^CA hep^* mice compared to Cre-negative controls (3.82 vs. 0.98%, *p <* 0.001; Figure 1G), which was also confirmed by quantification of hepatic hydroxyproline levels (77.6 vs. 173,7μg OH-proline/mg liver; Figure 1H). In line with these results, immunofluorescence staining for desmin revealed increased numbers of hepatic stellate cells in *Ctnnb1^CA hep^* mice in the portal tracts surrounding proliferating cholangiocytes suggesting hepatic stellate cells as the major source of activated myofibroblasts (5.93 vs. 2.08%, *p <* 0.0001; Supplementary Figure S1G). Thus, our findings indicate a chronic cholestatic liver injury with a biliary type of fibrosis and dedifferentiation of hepatocytes into cholangiocytes.

### *Ctnnb1^CA hep^* mice are characterized by severe cholestasis and qualitative changes in bile acid composition due to deregulation of enzymes involved in bile acid synthesis

**Figure 2:**
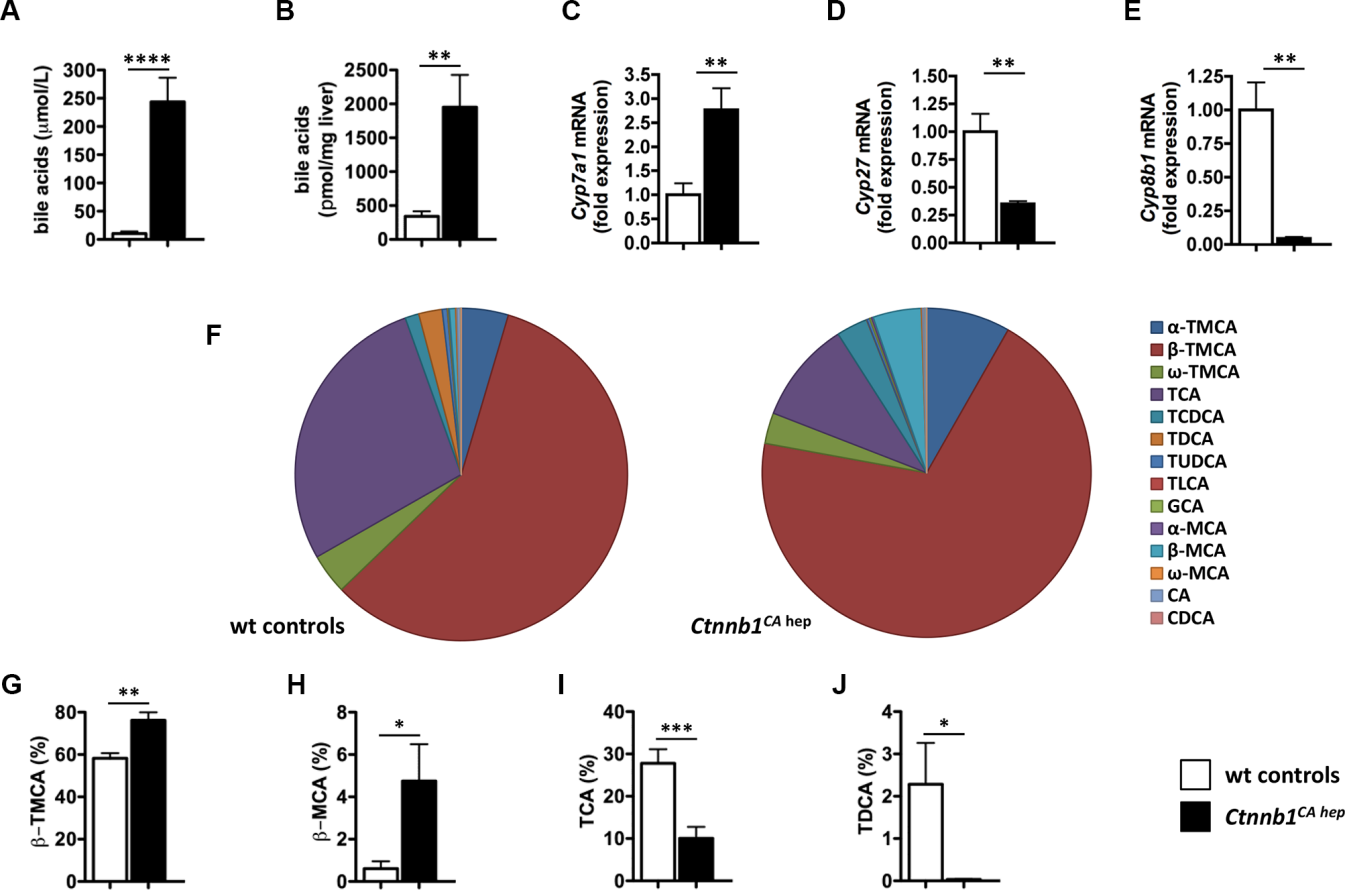
Ctnnb1CA hep mice are characterized by severe cholestasis and qualitative changes in bile acid composition *Ctnnb1^CA hep^* mice displayed significantly increased serum (**A**) and hepatic (**B**) bile acid levels. Expression of *Cyp7a1* (**C**) was significantly increased in *Ctnnb1^CA hep^* mice, whereas mRNA levels of *Cyp27* (**D**) and *Cyp8b1* (**E**) were decreased in livers with continuous β-catenin activation. The composition of the bile acid pool was altered in *Ctnnb1^CA hep^* mice (**F**). The percentage of tauro-β-muricholic acid (β-TMCA, **G**) and β-muricholic acid (β-MCA, **H**) of the total bile acid pool was significantly increased in *Ctnnb1^CA hep^* mice, whereas the percentage of taurine-conjugated cholic acid (TCA, **I**) and deoxycholic acid (TDCA, **J**) was decreased.

Electron microscopy revealed enlarged and dilated bile canaliculi in *Ctnnb1^CA hep^* mice (Supplementary Figure S2A). Remarkably, serum bile acid levels were ∼25-fold increased in *Ctnnb1^CA hep^* mice compared to controls (243.8 vs. 10.5 μmol/L, *p <* 0.0001; Figure 2A). Furthermore, bile acid levels were also significantly increased in livers of *Ctnnb1^CA hep^* mice compared to Cre-negative controls (1951.8 vs. 340.7 pmol/mg liver tissue, *p <* 0.01; Figure 2B). Next, we evaluated expression of Cytochrome P450 key enzymes involved in bile acid synthesis by qPCR to address if increased bile acid levels in *Ctnnb1^CA hep^* mice are the consequence of increased synthesis in hepatocytes. Expression of *Cyp7a1*, the first and rate-limiting enzyme of the classical pathway, was significantly increased in *Ctnnb1^CA hep^* mice (Figure 2C). In contrast, mRNA levels of *Cyp27* and *Cyp8b1*, key enzymes of the alternative pathway of bile acid synthesis, were significantly reduced in *Ctnnb1^CA hep^* mice (Figure 2D and 2E). Furthermore, *Cyp2b10*, which is a key enzyme in detoxification, was completely abolished in *Ctnnb1^CA hep^* (Supplementary Figure S2B). These results indicate that increased serum and tissue levels of bile acids in *Ctnnb1^CA hep^* mice result from activation of the classical pathway of bile acid synthesis.

Next we quantified individual bile acids in liver tissue of *Ctnnb1^CA hep^* mice and Cre-negative littermate controls to assess if differential expression of various CYPs involved in bile acid homeostasis translates into changes of bile acid composition. As absolute amounts of different bile acids were significantly increased in *Ctnnb1^CA hep^* mice, we analyzed the relative contribution of individual sterol intermediates to the total hepatic bile acid pool (Figure 2F). The percentage of tauro-β-muricholic acid (β-TMCA) and β-muricholic acid (β-MCA) was significantly increased in *Ctnnb1^CA hep^* mice compared to Cre-negative controls (β-TMCA: 76.22% vs. 58.25%, *p <* 0.01; β-MCA: 4.75% vs. 0.61%, *p <* 0.05; Figure 2G and 2H). In contrast, taurine-conjugated cholic acid (TCA) and deoxycholic acid (TDCA) were relatively decreased in livers of *Ctnnb1^CA hep^* mice compared to Cre-negative controls (TCA: 10.05% vs. 27.78%, *p <* 0.001; TDCA: 0.03% vs. 2.29%, *p <* 0.05; Figure 2I and 2J).

### Cholestasis causes up-regulation of compensatory bile acid transporters in livers, intestines and kidneys of *Ctnnb1^CA hep^* mice

**Figure 3:**
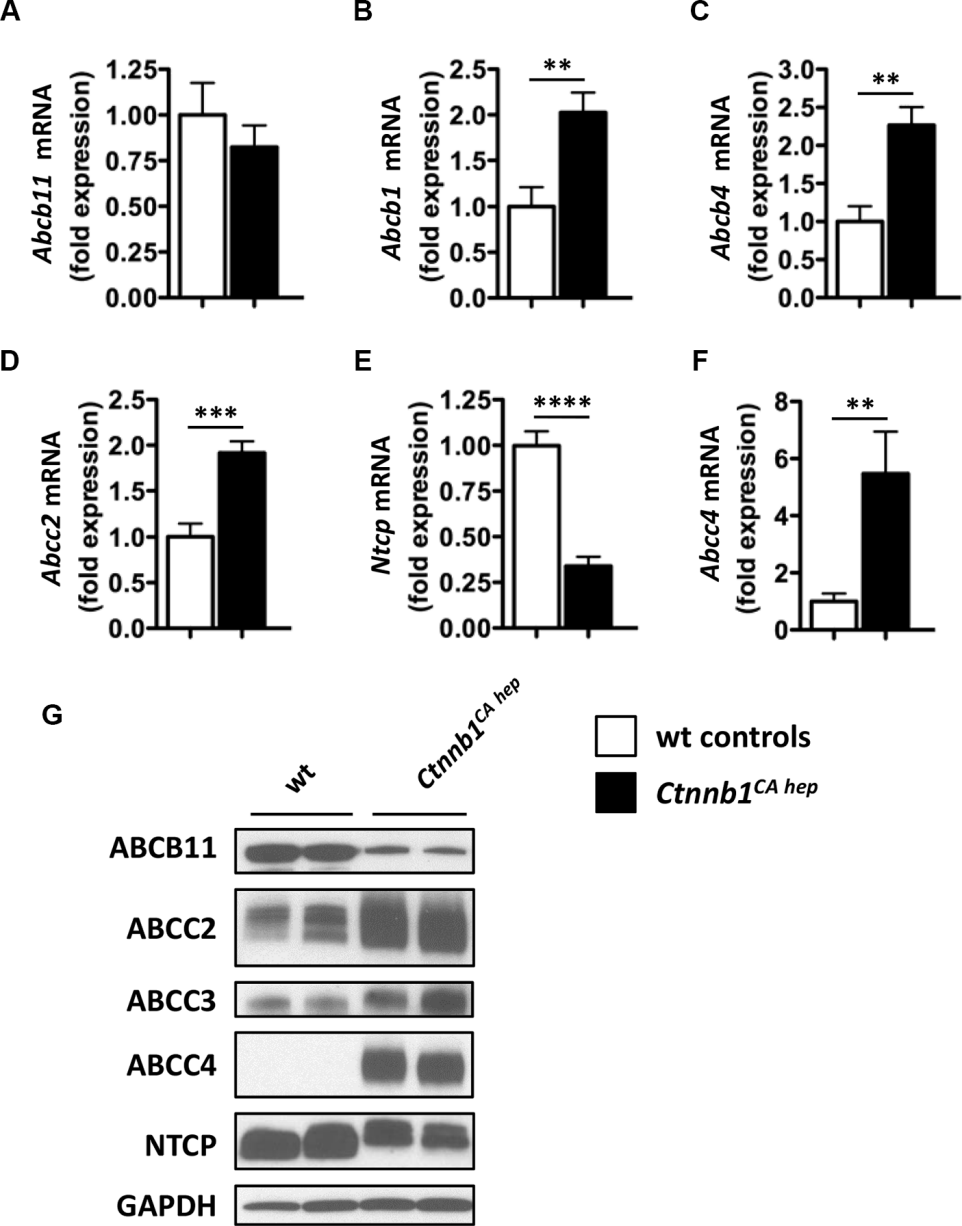
Ctnnb1CA hep mice display adaptive changes in expression of compensatory bile acid transporters The expression of *Abcb11* was not altered in *Ctnnb1^CA hep^* mice (**A**), whereas mRNA levels of compensatory bile acids transporters of *Abcb1* (**B**), *Abcb4* (**C**) and *Abcc2* (**D**) were significantly increased in *Ctnnb1^CA hep^* mice. Expression of *Ntcp* was decreased (**E**) and *Abcc4* increased in *Ctnnb1^CA hep^* mice (**F**). Deregulation of bile acid handling transporters was confirmed on protein level by Western blot analysis with indicated antibodies (**G**).

Due to high bile acid levels detected in serum and livers of *Ctnnb1^CA hep^* mice, we analyzed expression of various bile acid transporters, to test for putative changes in bile secretion, re-adsorption or excretion. *Ctnnb1^CA hep^* mice did not differ from controls with regards to expression of bile salt exporting pump (*Bsep*) also known as ATP-binding cassette transporter b11 (*Abcb11*) as evaluated by qPCR (Figure 3A). In contrast, mRNA levels of compensatory bile acid transporters including *Abcb1* (also known as multidrug resistance protein 1 (*Mdr1*)), *Abcb4* (also known as *Mdr2*), and *Abcc2* (also known as multidrug resistance-associated protein 2 (*Mrp2*)) were significantly increased in *Ctnnb1^CA hep^* mice (Figure 3B–3D). Consistent with increased expression of compensatory bile acid transporters at the apical lumen, *Ctnnb1^CA hep^* mice displayed significantly reduced mRNA levels of the Na^+^-taurocholate co-transporting polypeptide (*Ntcp*, also known as *Scl10a1*) (Figure 3E). Furthermore, *Ctnnb1^CA hep^* mice expressed significantly increased mRNA levels of *Abcc4* (also known as *Mrp4*) favoring excretion of bile acids from hepatocytes into the systemic circulation (Figure 3F). Expression of organic anion-transporting polypeptide (*Oatp*) 1 and 2 as well as organic solute transporter (*Ost*) α and β did not differ between *Ctnnb1^CA hep^* mice and controls (data not shown). Changes in expression of various transporters were confirmed at protein level (Figure 3G). In contrast to mRNA data, ABCB11 was reduced in *Ctnnb1^CA hep^* mice compared to controls. These results suggest reactive up-regulation of compensatory bile acid transporters in response to increased intracellular bile acid levels in hepatocytes. In line with this hypothesis down-regulation of NTCP at basolateral membranes prevents bile acids derived from the enterohepatic circulation from entering hepatocytes of *Ctnnb1^CA hep^* mice preventing further accumulation of bile acids in hepatocytes (Figure 3H).

**Figure 4:**
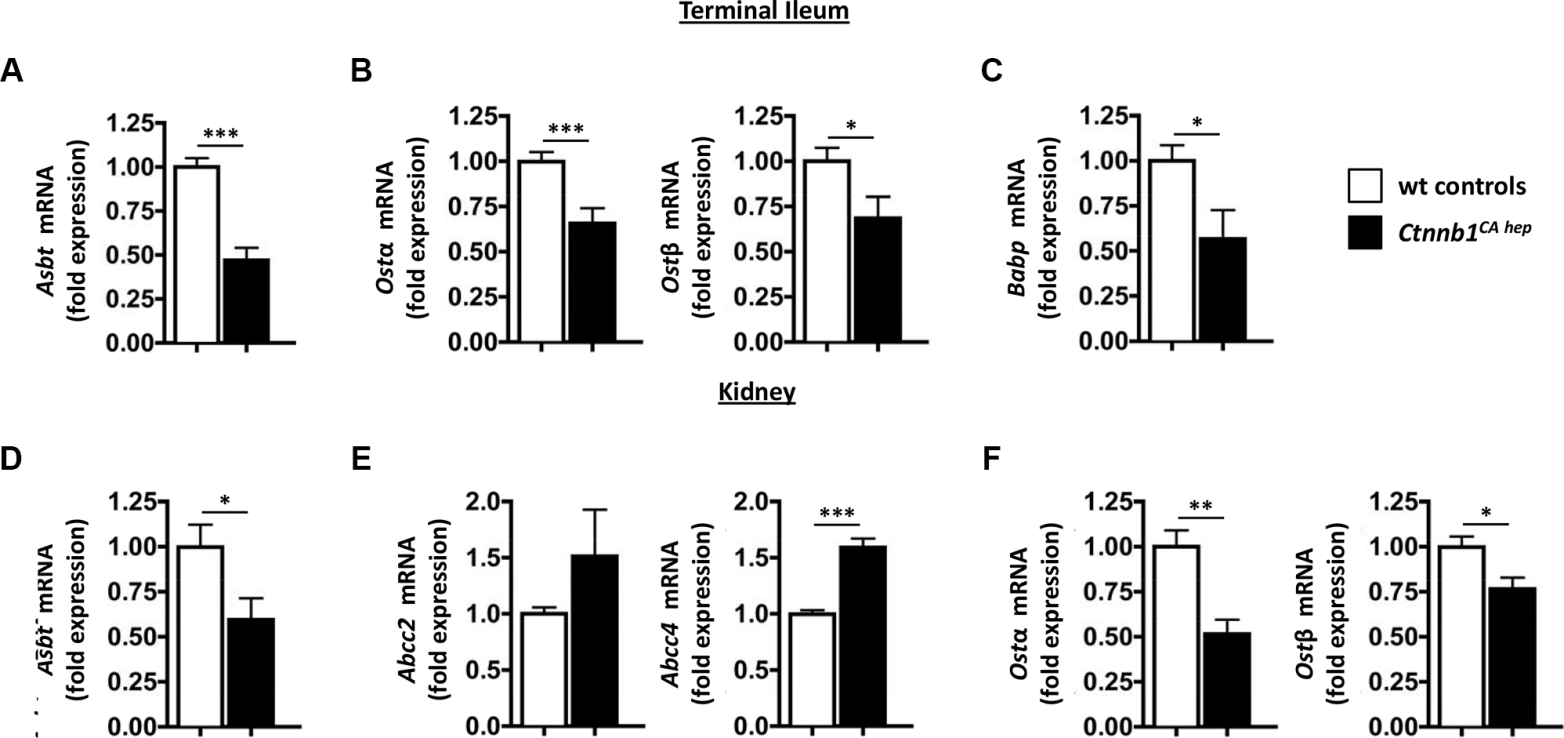
Cholestasis causes deregulation of compensatory bile acid transporters in intestines and kidneys of Ctnnb1CA hep mice Enterocytes in the terminal ileum of *Ctnnb1^CA hep^* mice displayed significantly reduced mRNA levels of the bile acid handling transporters *Asbt* (**A**), *Ost*α and β (**B**) and *Babp* (**C**). Proximal convoluted tubule cells in kidneys of *Ctnnb1^CA hep^* mice displayed significantly reduced mRNA levels of *Asbt* (**D**). In contrast, mRNA levels of *Abcc2* and *Abcc4* were increased in *Ctnnb1^CA hep^* mice (**E**). Expression of *Ost*α and β were significantly reduced in kidneys of *Ctnnb1^CA hep^* mice (**F**).

Under physiological conditions, bile acids are extensively recycled in the intestine and the kidneys in order to maintain a functional bile acid pool. Enterocytes in the terminal ileum express the apical sodium-dependent bile acid co-transporter (*Asbt*, also known as *Slc10a2*) at the luminal side for reabsorption of secreted bile acids and *Ost*α and *Ost*β at the basolateral membrane for efflux of bile acids into the portal circulation [31–33]. *Ctnnb1^CA hep^* mice expressed significantly reduced mRNA levels of *Asbt* as well as *Ost*α and *Ost*β compared to controls in enterocytes (Figure 4A and 4B). In line with reduced reabsorption of bile acids from the small intestine enterocytes of *Ctnnb1^CA hep^* mice expressed significantly lower mRNA levels of intestinal bile acid protein (*Babp*), an intracellular transport system for bile acids from the apical to the basolateral membrane of enterocytes (Figure 4C) [33].

Bile acids are also filtrated in the glomerulus and reabsorbed by epithelial cells of proximal convoluted tubules in the kidney. In parallel to enterocytes in the terminal ileum, proximal convoluted tubule cells express *Asbt* at the luminal membrane for reabsorption of filtrated bile acids [34]. *Asbt* mRNA levels were significantly decreased in kidneys of *Ctnnb1^CA hep^* mice compared to Cre-negative littermate controls (Figure 4D). In contrast, mRNA levels of *Abcc2* and *Abcc4* also located at the luminal membrane of proximal convoluted tubule cells but catalyzing transport of bile acids into the lumen were increased in *Ctnnb1^CA hep^* mice compared to Cre-negative littermate controls (Figure 4E). Proximal convoluted tubule cells also express *Ost*α and β at the basolateral membrane for efflux of bile acids into the systemic circulation. Similar to enterocytes in the terminal ileum, mRNA levels of *Ost*α and β were significantly reduced in kidneys of *Ctnnb1^CA hep^* mice compared to Cre-negative littermate controls (Figure 4F). In summary, these results suggest activation of compensatory transporter deregulation due to feedback mechanisms in the liver, intestine and kidneys of *Ctnnb1^CA hep^* mice favoring excretion of bile acids aiming to reduce the total bile acid pool. Expression levels of bile acid transporters also suggested that the high levels of bile acid were due to over-production rather than non-functional drainage.

To investigate the deregulation of bile acid homeostasis in more detail we examined the feedback mechanisms involved in the regulation of the bile acid pools including the enterokine fibroblast growth factor 15 (*Fgf15*), which is a critical regulator of bile acid homeostasis. Under physiological conditions, FGF15 is produced by enterocytes in response to FXR signaling due to increased intestinal bile acid levels and secreted into the portal blood stream. In the liver FGF15 binds to its receptor FGFR4 expressed by hepatocytes, which suppresses transcription of *Cyp7a1*. Thus, high intraluminal levels of bile acids in the intestine suppress bile formation through a negative feed back mechanism executed by FGF15 and FGFR4 [35].

**Figure 5:**
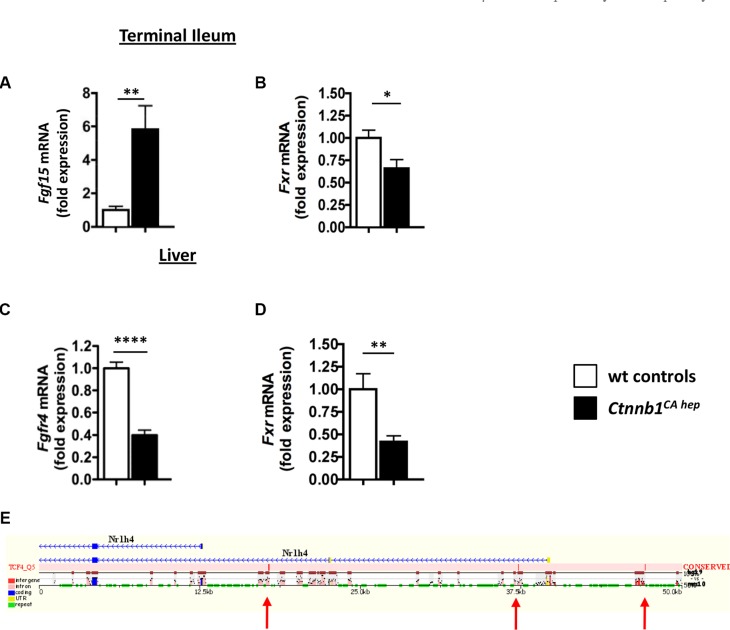
Deregulation of molecules involved in bile acid homeostasis in Ctnnb1CA hep mice Expression of the enterokine *Fgf15* was significantly increased in enterocytes of *Ctnnb1^CA hep^* mice (**A**), while *Fxr* was significantly down-regulated (**B**). The FGF15 responsive receptor *Fgfr4* was significantly decreased in the livers of *Ctnnb1^CA hep^* mice (**C**). mRNA levels of *Fxr* were also decreased in livers of *Ctnnb1^CA hep^* mice compared to controls (**D**). *In silico* screening for human/mouse conserved β-catenin binding sites using the ECR browser (http://ecrbrowser.dcode.org) identifies conserved binding sites in the promoter region of the FXR encoding gene *Nr1h4* (**E**). Arrows indicate the locations of the conserved TCF4 binding sites.

In line with increased bile formation and secretion into the small intestine, enterocytes of *Ctnnb1^CA hep^* mice expressed significantly increased *Fgf15* mRNA compared to Cre-negative littermate controls (Figure 5A). Intriguingly, levels of *Fxr* (*Nr1h4*) were significantly decreased in the terminal ileum (Figure 5B). In the liver, *Fgfr4* mRNA levels were significantly reduced in *Ctnnb1^CA hep^* mice (0.45 vs. 1.00, *p <* 0.01; Figure 5C). Additionally, *Fxr* (*Nr1h4*) expression, which can be directly activated by increased intra-hepatocellular bile acid levels repressing Cyp7a1 expression, was significantly down-regulated in the livers of *Ctnnb1^CA hep^* mice (Figure 5D). *In silico* analyses of liver transporters and molecules involved in feedback control of bile synthesis revealed conserved TCF4 binding sites within the *Fxr* gene, suggesting a direct effect of β-catenin on *Fxr* expression (Figure 5E). Together, these data suggest that continuous β-catenin activation results in disruption of the FXR-FGF15-FGFR4-CYP7A1 axis in *Ctnnb1^CA hep^* mice and loss of feedback control of bile acid production in the liver.

### Cholestasis in *Ctnnb1^CA hep^* mice is not a consequence of developmental abnormalities

**Figure 6:**
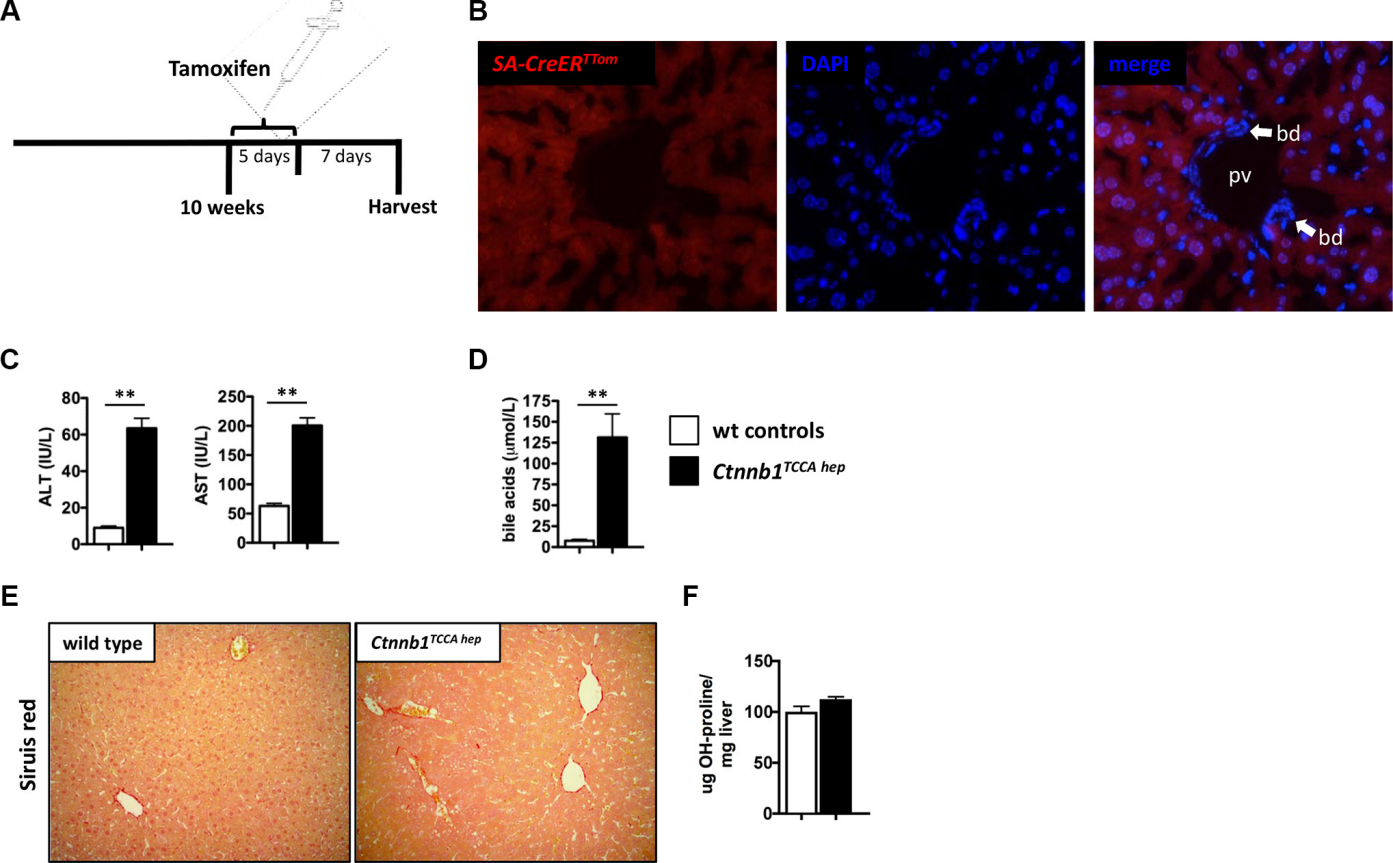
Cholestasis in Ctnnb1TCCA hep mice is not a consequence of developmental abnormalities Tamoxifen injection scheme for *SA-CreER^TTom^* and *Ctnnb1^TCCA hep^* mice (**A**). Tamoxifen administration leads to the activation of the Cre recombinase and consequently to expression of tdTomato (red fluorescence) in hepatocytes but not in cholangiocytes (counterstaining with DAPI, bd = bile duct, pv = portal vein) (**B**). Serum ALT, AST and bile acid levels were significantly increased in *Ctnnb1^TCCA hep^* mice (**C** and **D**). *Ctnnb1^TCCA hep^* mice display only minor signs of fibrosis (**E** and **F**).

β-catenin plays a crucial role during embryogenesis. *Albumin-Cre* mice express Cre recombinase as early as day 15.5 of embryonic development [36]. Accordingly, the phenotype of *Ctnnb1^CA hep^* mice might result from developmental abnormalities. To address this caveat we generated mice in which hepatocyte-specific expression of the Cre recombinase can be temporally controlled. To test the validity of the system we crossed *Serum albumin CreER^T2^* (*SA-CreEr^T2^*) mice, in which the expression of the Cre recombinase is inducible by tamoxifen, to a reporter mouse line in which the red fluorescent protein tdTomato is located after a loxP-flanked STOP cassette. Hence the offspring of this breeding (*SA-CreEr^TTom^*) express tdTomato only in recombinated cells after removal of the STOP cassette induced by tamoxifen injection. *SA-CreEr^TTom^* mice were treated with intra-peritoneal injections of tamoxifen at the age of 10 weeks for five consecutive days and sacrificed 7 days after the last injection (Figure 6A). Fluorescence microscopy revealed that tdTomato is only expressed in hepatocytes (counterstaining with DAPI) while cholangiocytes of bile ducts display no red fluorescence confirming that the SA-Cre recombinase is specifically active in hepatocytes (Figure 6B). Subsequently, we generated tamoxifen inducible *Ctnnb1^TCCA hep^* mice, by crossing *Ctnnb1* exon 3 floxed mice to *Serum albumin CreER^T2^* mice. Activity of Cre recombinase and thus recombination was controlled by intraperitoneal injection of tamoxifen according to the same injection schedule as for *SA-CreEr^TTom^* mice. Intriguingly, *Ctnnb1^TCCA hep^* mice displayed elevated *s*erum ALT and AST levels (ALT: 63.5 vs. 5.5 IU/L, *p <* 0.01; AST: 200.5 vs. 63 IU/L, *p <* 0.01; Figure 6C), as well as elevated bile acid (BA) levels (AP: 65 vs. 40 IU/L, *p <* 0.01; BA: 131.1 vs. 28.2 μmol/L, *p <* 0.01; Figure 6D). Only minor signs of fibrosis, as evaluated by Sirius red staining and hepatic hydroxyproline levels, could be detected, which might be due to the short period of β-catenin induction in this system (Figure 6E and 6F). Analogous to *Ctnnb1^CA hep^* mice, *Ctnnb1^TCCA hep^* mice displayed a reactive bile duct phenotype indicating cholestasis (Supplementary Figure S3A), with no signs of hyperproliferation (Supplementary Figure S3B) or apoptosis (Supplementary Figure S3C) as compared to control mice.

**Figure 7:**
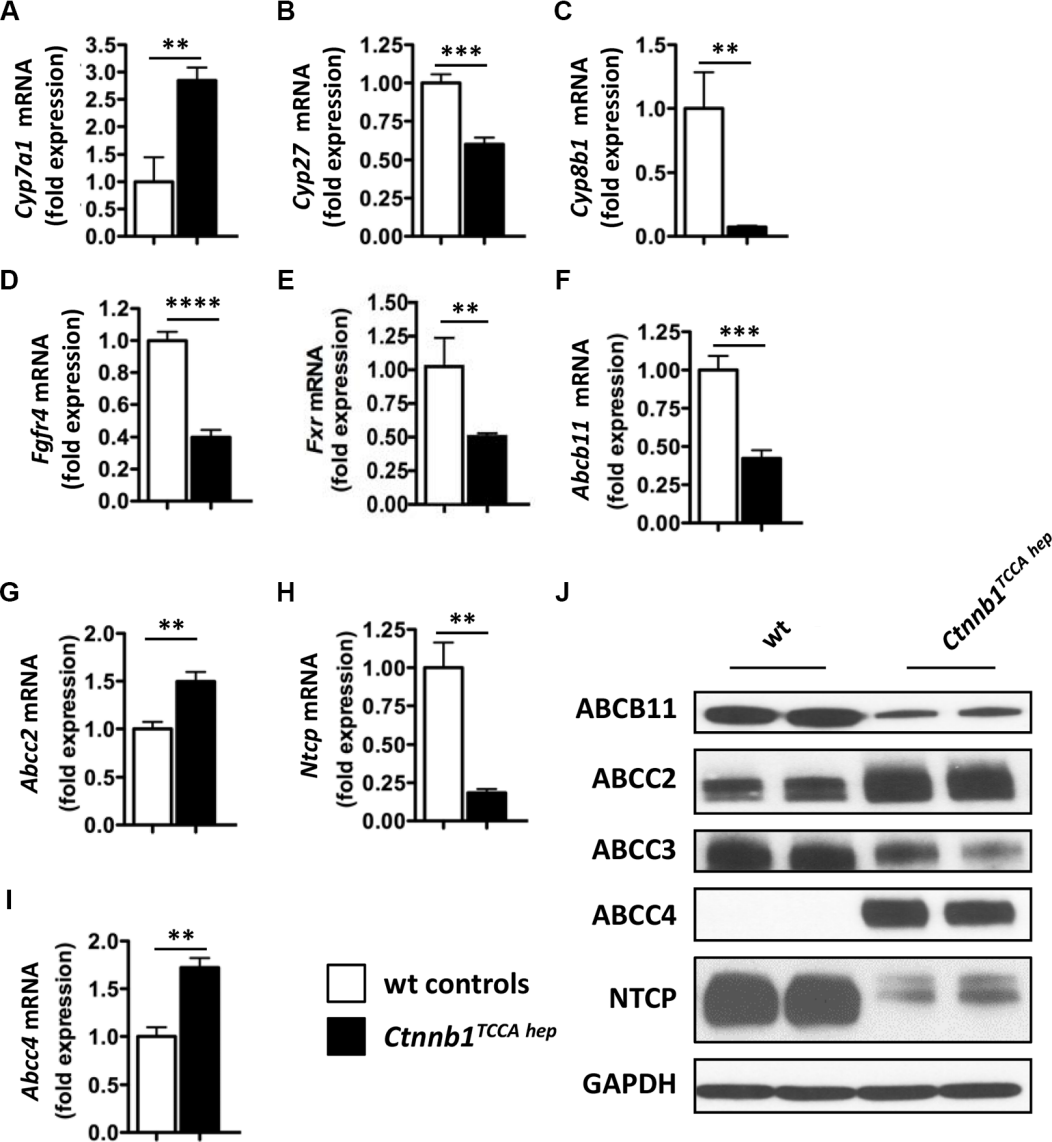
Ctnnb1TCCA hep mice display deregulation of key enzymes for bile acid synthesis and hepatic transport Expression of *Cyp7a1* (**A**) was increased in *Ctnnb1^TCCA hep^* mice, whereas mRNA levels of *Cyp27* (**B**), *Cyp8b1* (**C**), *Fgfr4* (**D**) and *Fxr* (**E**) were significantly reduced. mRNA levels of *Abcb11* were reduced (**F**) and of *Abcc2* increased in *Ctnnb1^TCCA hep^* mice (**G**). Downregulation of *Ntcp* (**H**) and upregulation of *Abcc4* (**I**) in livers with continuous β-catenin activation. Deregulation of bile acid transporters was confirmed at protein level by Western blot analysis with indicated antibodies (**J**).

Furthermore, *Ctnnb1^TCCA hep^* mice displayed the same expression pattern of enzymes involved in bile acid synthesis as *Ctnnb1^CA hep^* mice, including up-regulation of *Cyp7a1* and down-regulation of *Cyp27* and *Cyp8b1* (Figure 7A–7C) as well as downregulation of *Cyp2b10* (Supplementary Figure S3D). Additionally, *Fgfr4* was also significantly down-regulated in *Ctnnb1*TC*^CA hep^* mice (Figure 7D). Moreover, *Fxr* expression was significantly reduced in livers as well as in the terminal ileum of *Ctnnb1^TCCA hep^* mice (Figure 7E and Supplementary Figure S3E), providing further evidence for decoupling of the FXR-FGF15-FGFR4-CYP7A1 axis in response to continuous β-catenin signaling. *Ctnnb1*TC*^CA hep^* mice were also characterized by deregulation of hepatic bile acid transporters similar to *Ctnnb1^CA hep^* mice. *Abcb11* mRNA levels were significantly reduced in livers of *Ctnnb1^TCCA hep^* mice, whereas expression of *Abcc2* was increased (Figure 7F and 7G). Consistent with results observed in *Ctnnb1^CA hep^* mice, *Ctnnb1*TC*^CA hep^* mice displayed significantly reduced mRNA levels of *Ntcp* (Figure 7H) and increased mRNA levels of *Abcc4* (Figure 7I). The expression of hepatic transporters was also quantified on protein level, where the same trend as in the qPCR analyses was observed (Figure 7J).

In summary, these results suggest that severe cholestasis in *Ctnnb1^CA hep^* mice is a direct consequence of continuous β-catenin signaling and not related to potential disturbances of liver architecture as a consequence of genetic recombination during embryonic development.

## DISCUSSION

β-Catenin signaling has been associated both with liver development, homeostasis and disease. Activation of the Wnt/β-catenin pathway is frequently observed in HCC, and missense mutations in exon 3 of *Ctnnb1* exhibit a histologically more aggressive phenotype and are associated with severe cholestasis, vascular invasion and recurrence of disease after orthotopic liver transplantation [24, 37]. In line with data obtained from mice it was suggested that cholestasis is a marker of HCCs with activating β-catenin mutations [38]. Aside from its well-established role in diverse neoplastic diseases of the liver, active β-catenin signaling has been implicated in hepatic fibrosis important for stellate cell activation, and NASH, highlighting its role for the regulation of metabolic processes in the liver [27].

In the present study we generated a mouse model to investigate the consequences of continuous β-catenin signaling in hepatocytes on liver biology. Using two mouse models of fetal and post-natal β-catenin activation we observed a cholestatic phenotype within days of β-catenin activation. A hallmark of both mouse models were the enormously increased bile acid levels in serum and livers compared to Cre-negative controls. Although *Ctnnb1^CA hep^* mice display severely disturbed liver architecture and biliary type of fibrosis our data suggest no defects in bile drainage due to obstipation, because *Ctnnb1*TC*^CA hep^* mice also suffer from cholestasis and highly elevated bile acid levels without prominent liver pathology and fibrosis. Additionally, the striking deregulation of molecules involved in bile acid synthesis pathways provides evidence for accelerated *de novo* synthesis of bile acids rather than an anatomical cause of cholestasis. Our data are in line with studies using conditional *Apc* knockouts to induce stable β-catenin expression in hepatocytes that demonstrate defects in liver zonation and a positive effect of activated Wnt/β-catenin signaling on bile acid metabolism and transport as well as cholesterol and drug metabolism [10, 39]. In one of these studies, upregulation of *Cyp7a1*, *Cyp27a1* and *CAR* in liver from *Apc* knockout mice with constitutively activated β-catenin was reported and suggested that *Cyp27a1* but not *Cyp7a1* is a direct target of β-catenin [10]. In contrast, we report significant downregulation of *Cyp27a1* in *Ctnnb1^CA hep^* and *Ctnnb1^TCCA hep^* mice. Furthermore, the expression pattern of enzymes and transporters involved in bile acid formation and secretion argues against a strictly CAR-mediated adaptive response in *Ctnnb1^CA hep^* and *Ctnnb1*TC*^CA hep^* mice.

Additionally, it was previously shown that *Ctnnb1^Δhep^* mice are protected from acetaminophen induced hepatotoxicity due to lack of CYP2E1 expression [19]. The authors also reported reduced expression of *Cyp1a2* and *Cyp2c29* but unchanged expression of *Cyp1a1* and *Cyp3a11* in livers of *Ctnnb1^Δhep^* mice suggesting differential rather than global regulation of CYP450 isoforms by β-catenin. Direct transcriptional regulation of particular CYP450 isoforms by β-catenin is further supported by data obtained in mice with liver tumors with activating β-catenin mutations [40]. In this respect, it was demonstrated that glutamine synthetase positive tumors express increased levels of CYP1A, CYP2B, CYP2C and CYP2E1. Furthermore, the authors provided evidence for direct activation of CYP2B1 by β-catenin using luciferase assays and amplification of 2,3,7,8-tetrachlordibenzop-dioxin mediated activation of a dioxin-responsive reporter gene assay by β-catenin [40].

Bile acid synthesis is subject to a tightly controlled feedback mechanism under physiological conditions, depending on bile acid levels in the liver and in the ileum. We observed significantly increased mRNA levels of the FXR-dependent enterokine FGF15 in enterocytes of *Ctnnb1^CA hep^* mice. However, FXR levels were significantly decreased in the terminal ileum and in livers of *Ctnnb1^CA hep^* mice. Despite high FGF15 levels in enterocytes, Fgfr4 expression was reduced in hepatocytes of *Ctnnb1^CA hep^* mice. We speculate that β-catenin might directly interfere with the bile acid feedback loop by downregulation of *Fxr* expression. An inverse correlation of FXR and β-catenin expression was previously observed in HCC and *Fxr* knockout mice develop HCC through activation of Wnt/β-catenin signaling [41].

In our model, cholestasis in *Ctnnb1^CA hep^* mice was associated with compensatory up- and down-regulation of accessory bile acid transporters in hepatocytes as well as in enterocytes in the terminal ileum and in epithelial cells of proximal convoluted tubules in the kidney. We observed increased expression of *Abcc4* in livers of *Ctnnb1^CA hep^* mice suggesting activation of a Constitutive Androstane Receptor (CAR)-mediated protection pathway leading to secondary detoxification of bile acids. However, expression of *Cyp2b10*, another classical CAR target gene, was completely abolished in *Ctnnb1^CA hep^* and *Ctnnb1*TC*^CA hep^* mice. These results indicate that up-regulation of enzymes and transporters involved in bile acid detoxification is not strictly CAR mediated and suggest a more complex mechanism in the induction of phase I, II and III detoxification systems in hepatocytes with continuous β-catenin signaling.

In contrast to our results, it was previously demonstrated that *Ctnnb1^Δhep^* mice display bile canalicular abnormalities, bile secretory defects and intrahepatic cholestasis [11]. However, bile acid levels in livers and serum were only modestly increased (∼2-3-fold) suggesting that observed cholestasis is the consequence of anatomical and structural abnormalities in *Ctnnb1^Δhep^* mice.

In summary, our study provides further evidence linking β-catenin signaling to deregulations of individual CYP450 isoforms, bile acid formation and therefore cholestatic liver disease. Our data further suggest that hepatocytes with continuous β-catenin signaling as observed in a significant subgroup of patients with HCC is associated with fibrosis due to cholestasis and accompanying changes in expression of various complementary bile acid transporters. Given the importance of different CYP450 isoforms and transporters for inactivation and export of various widely clinically used anticancer drugs our study improves our understanding regarding resistance of HCC with activating β-catenin mutations against cytotoxic therapy. Further studies are necessary, to understand whether the observed hepatic injury is a result of bile acid accumulation or a direct consequence of β-catenin activation. Additionally, it will be important to study the functionality of the bile secretory system in more detail in affected mice and to investigate whether bile duct proliferation is a primary phenotype or rather a regenerative response to chronic hepatocellular damage.

## MATERIALS AND METHODS

### Mice

Albumin-Cre mice (C57BL/6-Tg(Alb-cre)21Mgn/J) were obtained from the Jackson laboratories [42]. Mice in which exon 3 of the β-catenin gene is flanked by loxP sites were described previously [43]. Mice with a loxP flanked exon 3 of the *Ctnnb1* gene were crossed to *Albumin-Cre* mice to obtain mice with hepatocyte specific expression of a dominant stable form of β-catenin *(Ctnnb1^CA hep^* mice). *Ctnnb1^CA hep^* mice as well as their wild type littermates, which served as controls were sacrificed and analyzed at the age of 21 days.

*Serum albumin CreER^T2^* (*SA-CreER^T2^*) mice were described previously [44]. The expression of Cre recombinase in these mice is also under control of an *Albumin* promoter. In contrast to *Albumin-Cre* mice, the Cre recombinase protein is fused to the ligand-binding domain of a mutated estrogen receptor (Cre-ERT^2^). The Cre-ERT^2^ fusion protein is sequestered in the cytoplasm but undergoes a conformation change in the presence of 4-hydroxy-tamoxifen and is then able to migrate to the nucleus removing loxP flanked DNA sequences. The resulting offspring are termed *Ctnnb1* temporally controlled constitutive active (TCCA) in hepatocytes (*Ctnnb1^TCCA hep^*) mice. Adult mice at the age of 10 weeks received intra-peritoneal injections of tamoxifen (10 μg/g body weight, Sigma Aldrich) for five consecutive days and were sacrificed and analyzed 7 days after the last injection. Cre-negative littermates receiving identical treatment as Cre-positive animals were exclusively used as controls throughout the entire study. All experiments performed in this study were approved by the local ethical committee (BMWFW-66.009/0134-WF/V/3b/2015).

### Serum parameters

Serum parameters were quantified using standard methods or as described previously [45, 46].

### Histology

The left liver lobe was cut in the middle and used for histology. Tissue was fixed in 10% formalin over night at room temperature and subsequently embedded in paraffin. Five μm thick sections were cut and used for staining. Sirius red staining was performed as described previously [47]. Immunofluorescence staining for desmin was performed as described previously [48]. Immunofluorescence staining for Keratin 19 was performed using TROMA-III antibody (1:500, Developmental Studies Hybridoma Bank) using snap frozen liver tissue following fixation in 100% acetone for 10 minutes at -20 degree. Detailed protocols for each staining will be provided upon request.

### Quantitative real-time PCR

mRNA was isolated using TRI^®^ Reagent RNA Isolation Reagent (Sigma Aldrich) following the instructions of the manufacturer. Complementary DNA (cDNA) was generated using the High Capacity cDNA Reverse Transcription Kit (Applied Biosystems) following the instructions of the manufacturer. iQ™ SYBR^®^ Green Supermix (BioRad) was used for quantitative PCR (qPCR). Primer sequences were obtained from the qPrimerDepot (http://mouseprimerdepot.nci.nih.gov) and will be provided upon request. Melting curve analysis and agarose gel electrophoresis was performed to assess the quality of primers and the qPCR.

### Immunoblotting

Protein extracts of liver samples were prepared using RIPA buffer and protein concentration was determined using a BCA assay (Pierce). Western blotting was performed using standard methods as described previously [47, 49, 50].

### Bile acid quantification

Bile acids were quantified as described previously [51].

### Image J

Morphometric quantification of images was performed using Image J software (Image J 1.48f, National Institutes of Health).

### *In silico* screening

In silico screening was performed in https://ecrbrowser.dcode.org/

## SUPPLEMENTARY MATERIALS FIGURES


