# Supplementary material for: Hepatocyte specific expression of an oncogenic variant of β-catenin results in cholestatic liver disease
Source: Oncotarget. 2016 Nov 23;7(52):86985–98. doi: 10.18632/oncotarget.13521 (PMC5349966; doi:10.18632/oncotarget.13521)
Supplement: Supplementary file 1 [file oncotarget-07-86985-s001.pdf]

Hepatocyte specific expression of an oncogenic variant of β-catenin results in cholestatic liver disease

Supplementary Materials

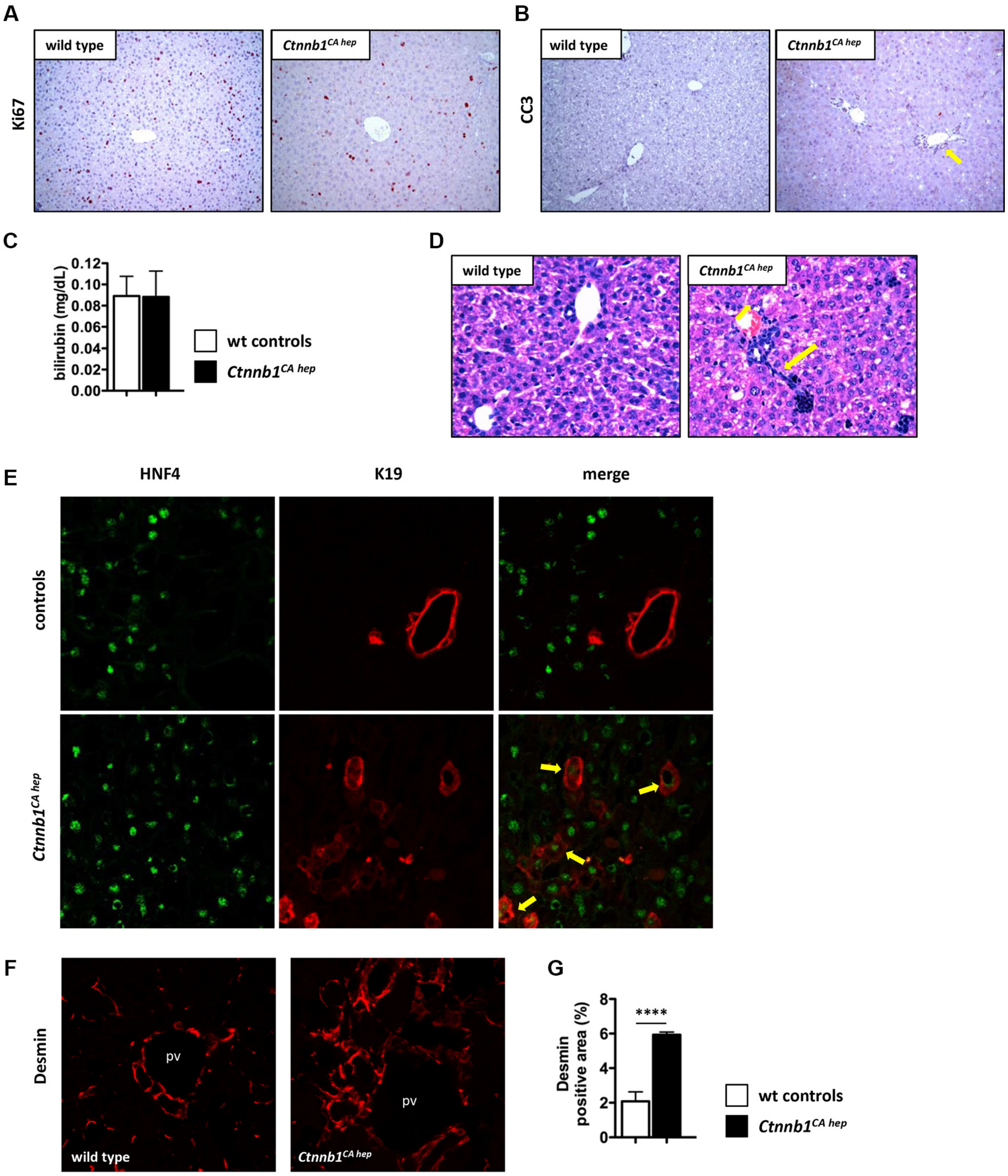

Supplementary Figure S1: Histological analysis of livers of *Ctnnb1$^{CA\ hep}$* mice. Livers of *Ctnnb1$^{CA\ hep}$* mice show no changes in proliferation compared to wt controls as analyzed by immunohistochemical staining for the proliferation marker Ki67 (**A**). Cholangiocytes of bile ducts reveal apoptosis in *Ctnnb1$^{CA\ hep}$* mice as detected by immunohistochemical staining for cleaved caspase 3 (CC3) (**B**). No changes in bilirubin could be detected (**C**). Increased cholangiocyte numbers are detected in H&E stained sections of *Ctnnb1$^{CA\ hep}$* mice compared to wt controls (yellow arrows) (**D**). *Ctnnb1$^{CA\ hep}$* mice displayed a dedifferentiation of hepatocytes, confirmed by double immunflourescence staining for HNF4 and Keratin 19 (K19). Double-positive cells are marked by arrows (**E**). *Ctnnb1$^{CA\ hep}$* mice show significantly enhanced numbers of collagen producing hepatic stellate cells as analyzed by immunofluorescence staining for Desmin (**F**). Desmin positive areas were significantly increased as evaluated by morphometric quantification using Image J (pv = portal vein) (**G**).

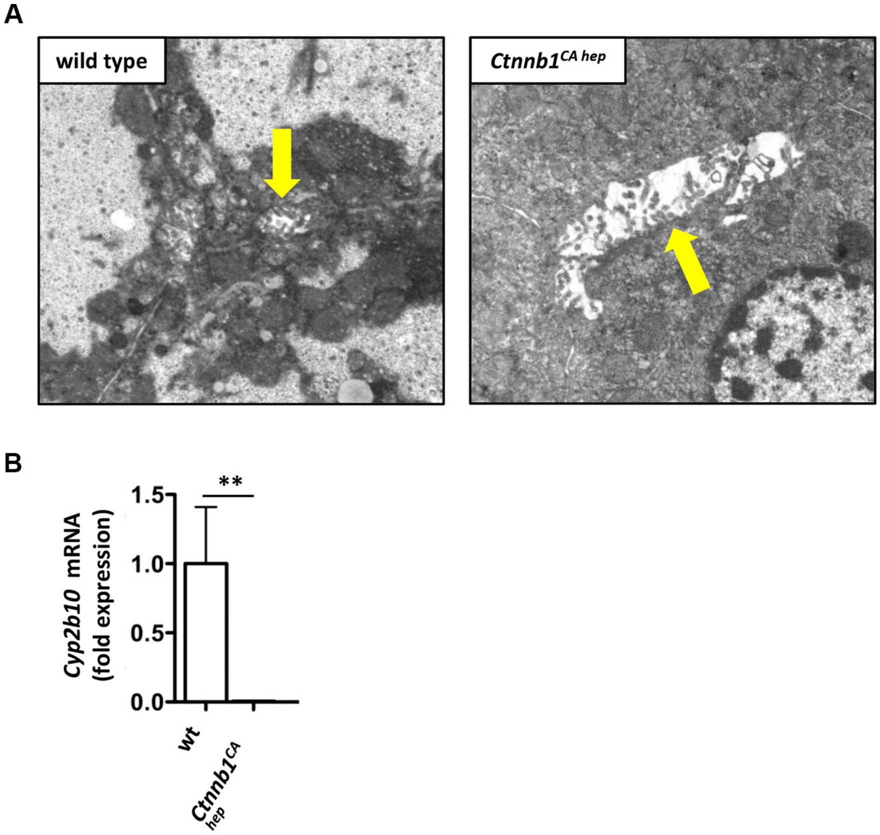

Supplementary Figure S2: Evaluation of electron microscopy images revealed dilated bile canaliculi in *Ctnnb1$^{CA\ hep}$* mice (yellow arrow) (A). mRNA levels of the CAR target gene *Cyp2b10* was completely abolished in livers of *Ctnnb1$^{CA\ hep}$* mice (**B**).

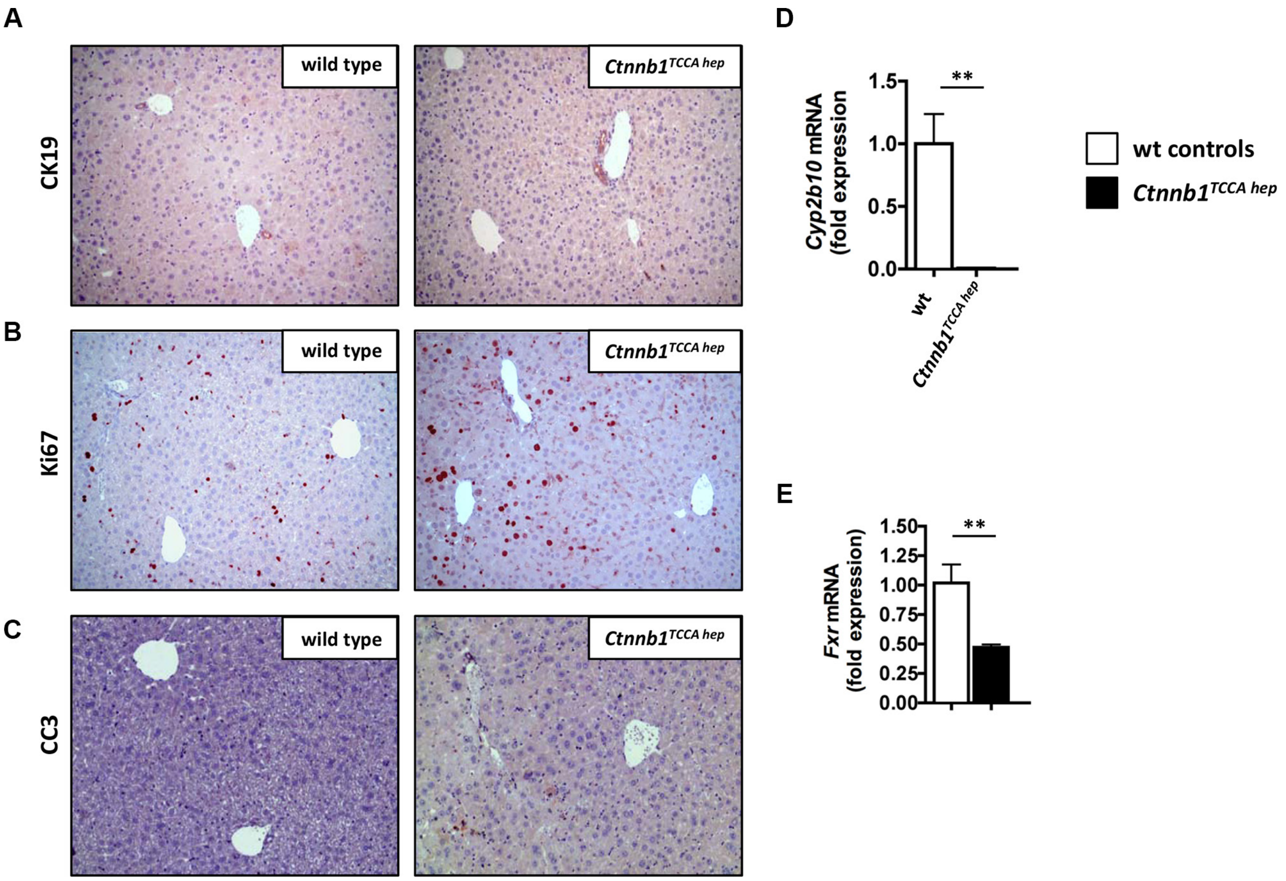

Supplementary Figure S3: *Ctnnb1$^{TCCA\ hep}$* mice display increased numbers of Keratin 19 positive cholangiocytes compared to wt controls. (**A**) Immunohistochemical staining for Ki67 and cleaved caspase 3 (CC3) revealed no significant changes in regards to proliferation (**B**) or apoptosis (**C**) in livers of *Ctnnb1$^{TCCA\ hep}$* mice. mRNA levels of the CAR target gene *Cyp2b10* was completely abolished in livers of *Ctnnb1$^{TCCA\ hep}$* mice (**D**). *Ctnnb1$^{TCCA\ hep}$* mice displayed a significant decrease of *Fxr* levels in enterocytes of the terminal ileum compared to wt controls (**E**).
